# Preparation and Laser-Induced Thermoelectric Voltage Effect of Bi_2_Sr_2_Co_2_O_y_ Thin Films Grown on Al_2_O_3_ (0001) Substrate

**DOI:** 10.3390/ma16145165

**Published:** 2023-07-22

**Authors:** Ping Zou, Dan Lv, Hui Zhang, Zhidong Li

**Affiliations:** 1 School of Materials Science and Engineering, Guizhou Minzu University, Guiyang 550025, China; 2 School of Materials Science and Engineering, Kunming University of Science and Technology, Kunming 650093, China

**Keywords:** Bi_2_Sr_2_Co_2_O_y_ thin films, pulsed laser deposition, deposition process, laser-induced thermoelectric voltage

## Abstract

Bi_2_Sr_2_Co_2_O_y_ thin films were grown on 10° vicinal-cut Al_2_O_3_ (0001) single crystalline substrates by pulsed laser-deposition techniques with in situ annealing, post-annealing and non-annealing process, respectively. The pure phase Bi_2_Sr_2_Co_2_O_y_ thin film was obtained with a non-annealing process. The result of X-ray diffraction showed that Bi_2_Sr_2_Co_2_O_y_ thin film was obviously *c*-axis preferred orientation. The laser-induced thermoelectric voltage signals were detected in Bi_2_Sr_2_Co_2_O_y_ thin films, which originated from the anisotropy of the Seebeck coefficient. The maximum peak value of laser-induced thermoelectric voltage was strong and could reach as large as 0.44 V and the response time was 1.07 μs when the deposition time was 6 min. Furthermore, the peak voltage enhanced linearly with the single-pulse laser energy. These characteristics demonstrate that Bi_2_Sr_2_Co_2_O_y_ thin film is also an excellent choice for laser energy/power detectors.

## 1. Introduction

In the 21st century, the application of thermoelectric thin films in optical and thermal radiation detectors has become the focus of research. Since the first discovery of laser-induced thermoelectric voltage (LITV) effect in high-temperature superconductor YBa_2_Cu_3_O_7-δ_ (YBCO) thin films [1,2,3], it has attracted much attention. The mechanism of this effect can be explained by the atomic layer thermopile model based on the anisotropic Seebeck effect [4,5]. In addition to a high-temperature superconductor, most research on LITV effect has been carried out in colossal magnetoresistance thin films and ferroelectric films, such as La_1−x_Sr_x_CoO_3_ (LSCO) [6,7], La_1−x_Sr_x_MnO_3_ (LSMO) [8], La_1−x_Sr_x_NiO_3_ (LSNO) [9], La_1−x_Ca_x_MnO_3_ (LCMO) [10,11,12,13], SrTi_1−x_Nb_x_O_3_ [14], Pb(Zr_0.3_Ti_0.7_)O_3_ [15], Pb(Mg_1/3_Nb_2/3_)O_3_-PbTiO_3_ [16] and so on. 

In recent years, cobalt-based oxides such as NaCo_2_O_4_ (NCO), Bi_2_Sr_2_Co_2_O_y_ (BSCO) and Ca_3_Co_4_O_9_ (CCO) have attracted much attention because of their good thermoelectric properties [17,18,19,20,21,22,23]. It is found that the difference in the Seebeck coefficient in the ab-plane and along the c-axis is very large through detailed studies on the electrical transport performance of above cobalt-based oxides [24]. It means that these cobalt-based oxides can be applied not only in the field of thermoelectric apparatus, but also in photoelectric sensors. Although research on the LITV effect in NCO and CCO thin films has been reported [25,26], it is rarely reported in BSCO thin film fabricated by pulsed laser-deposition (PLD) technique. Therefore, it is significant to study the LITV effect in BSCO thin film. In this paper, we focus on the LITV effect in BSCO thin films which are deposited on 10° titling Al_2_O_3_ (0001) substrate prepared by the PLD technique. As will be shown, the peak voltage value is 0.44 V and the response time is 1.07 μs, suggesting that this thin film is an important candidate material for laser energy/power meter.

## 2. Experiments

A BSCO polycrystalline ceramic target was prepared through a solid-state-reaction method using high-purity powder Bi_2_O_3_ (99.99 wt.%), SrCO_3_ (99.99 wt.%) and Co_2_O_3_ (99.99 wt.%). The Bi_2_O_3_ should be slightly excess in the process of a solid-state reaction as Bi could be slightly lost. These raw materials were mixed in an agate mortar for 1 h, then cold-pressed into a disk (the pressure is 20 MPa), sintered at 890 °C for 4 h in air and cooled down to room temperature. BSCO thin films were grown on Al_2_O_3_ (0001) substrates through the PLD technique. In order to find the best preparation process to obtain pure-phase BSCO thin films, in situ annealing, post-annealing and non-annealing were adopted, respectively. The ultraviolet (UV) pulsed laser beam from a KrF excimer laser (Lambda Physik, LPX300, Göttingen, Germany) was used to ablate the target. The wavelength, laser energy and repetition rate were 248 nm, 300 mJ and 5 Hz, respectively. The distance between the target and the substrate was 50 mm.

The crystal structure of BSCO thin films was characterized by X-ray diffraction (XRD, BDX3200, Peking University Instrument Factory, Beijing, China). An oscilloscope (Tektronix TDS210, Beaverton, OR, USA) was adopted to measure the LITV signals of BSCO thin films. In order to detect the LITV signals, two in-plane indium electrodes were placed on the thin film’s surface. The UV-pulsed laser beam with a wavelength of 248 nm was used as the thermal source to heat the thin film’s surface. The LITV signals were recorded when the UV laser irradiated the thin film’s surface.

## 3. Results and Discussion

### 3.1. In Situ Annealing

**Figure 1:**
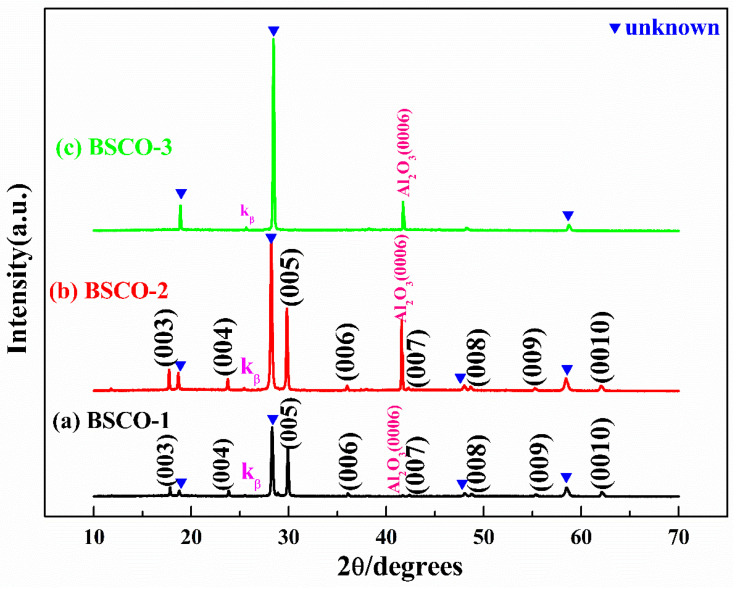
XRD patterns of thin films prepared by in situ annealing: (**a**) BSCO-1, (**b**) BSCO-2 and (**c**) BSCO-3.

Table 1 shows the preparation process of BSCO thin films by in situ annealing. Figure 1a–c are the XRD patterns of BSCO-1~BSCO-3. From Figure 1a, it can be seen that there are unknown diffraction peaks, and the intensity of the unknown diffraction peak in front of (005) is very strong. In order to research the effect of oxygen pressure on the phase of BSCO thin film, BSCO-2 thin film is prepared by reducing the oxygen pressure from 100 Pa to 60 Pa. As shown in Figure 1b, the phase of BSCO-2 thin film is similar to that of BSCO-1 thin film, the intensity of the unknown diffraction peak in front of (005) is still very strong. This shows that the oxygen pressure is not the main factor affecting the phase of BSCO thin film, and the substrate temperature should be the key factor affecting the phase of BSCO thin film. In the following experiment, the oxygen pressure is maintained at 60 Pa. When the oxygen pressure is low, the ions or atomic groups evaporated from the target surface are less likely to collide and have a large surface migration energy when reaching the substrate surface, which is conducive to the formation of nuclei and grain growth. Therefore, the oxygen pressure is selected as 60 Pa. The substrate temperature has a great influence on the phase of the thin films. Appropriate substrate temperature can make the adsorbed atoms have a certain kinetic energy, the atoms are easy to diffuse and migrate on the substrate surface, which makes the film easy to crystallize. At the same time, the internal stress of the film will also be reduced. BSCO-3 thin film is prepared by increasing the substrate temperature from 820 °C to 850 °C to find the appropriate substrate temperature. It can be seen from Figure 1c that, except for the (0006) diffraction peak of substrate Al_2_O_3_, all the diffraction peaks are basically unknown. This is mainly due to the decomposition of the film at high temperatures. 

### 3.2. Post-Annealing

**Figure 2:**
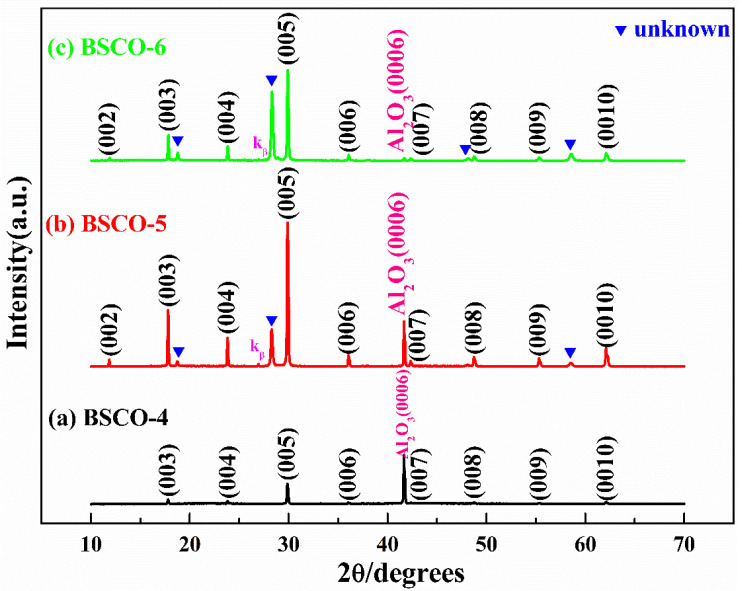
XRD patterns of thin films prepared by post-annealing: (**a**) BSCO-4, (**b**) BSCO-5 and (**c**) BSCO-6.

It can be known from the BSCO-1~BSCO-3 thin films that the single-phase BSCO thin film cannot be prepared by in situ annealing process. In order to prepare the single-phase BSCO thin film, the deposition process is adjusted, and the post-annealing process is adopted. Table 2 shows the deposition process of BSCO thin films by post-annealing. Figure 2a–c are the XRD patterns of BSCO-4~BSCO-6. From Figure 2a, it can be seen that the substrate temperature of 760 °C, annealing temperature of 510 °C and annealing time of 1 h are feasible. All impurity phases have been eliminated, but the intensity of the BSCO diffraction peak is weak, indicating that the crystallization quality of the thin film needs to be improved. Meanwhile, the lower limit temperature of a substrate during the deposition can be determined to be 760 °C. BSCO-5 thin film is prepared by increasing the substrate temperature from 760 °C to 800 °C. From Figure 2b, it can be seen that when the substrate temperature increased, the crystallization quality of the thin film is greatly improved, and the intensity of the unknown diffraction peak in front of (005) is also significantly lower than that of the BSCO-1~BSCO-3, indicating that the post-annealing process is effective for eliminating the impurity phase. BSCO-6 thin film is prepared by increasing the substrate temperature to 805 °C. As shown in Figure 2c, the intensity of the unknown diffraction peak in front of (005) is much stronger than that of the BSCO-5 thin film, indicating that under the same annealing temperature and annealing time, the substrate temperature of 800 °C is more suitable, and the higher substrate temperature cannot be selected to obtain the pure-phase thin film, that is, the upper limit temperature of the substrate is 800 °C. 

**Figure 3:**
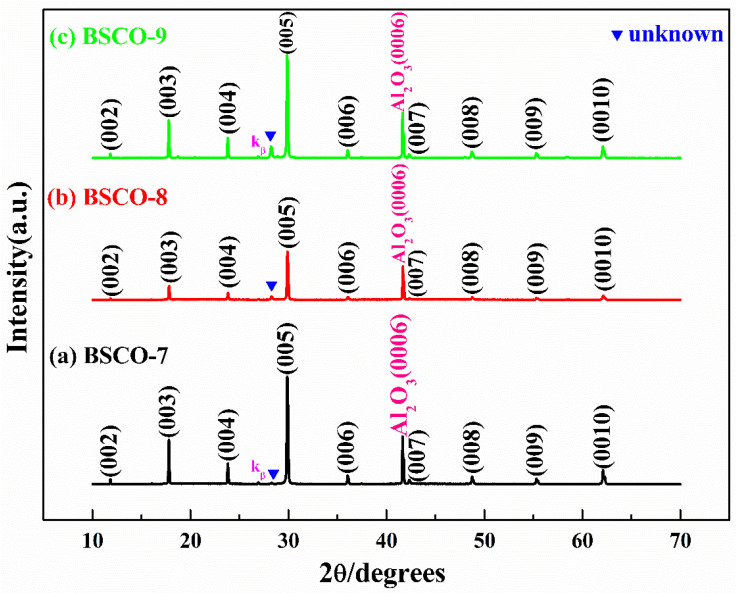
XRD patterns of thin films prepared by post-annealing: (**a**) BSCO-7, (**b**) BSCO-8 and (**c**) BSCO-9.

Figure 3a–c are the XRD patterns of BSCO-7~BSCO-9. The BSCO-7~BSCO-9 thin films also adopt the post-annealing process, and the annealing temperature is still kept at 510 °C, but the annealing time is extended to 2 h to research whether the impurity phase can be eliminated by extending the annealing time. It can be seen from Figure 3a that when the annealing time is extended to 2 h, other unknown diffraction peaks have been eliminated except the unknown diffraction peak in front of (005), and the intensity of the unknown diffraction peak in front of (005) is further reduced compared with that of BSCO-5, indicating that extending the annealing time is effective to eliminate the impurity phase. The BSCO-8 thin film is prepared by increasing the substrate temperature to 795 °C. From Figure 3b, it can be seen that when the substrate temperature rises from 780 to 795 °C, the intensity of the unknown diffraction peak in front of (005) enhanced, indicating that the deposition process of BSCO-7 is a better process under the same annealing condition. As shown in Figure 3c, when the substrate temperature rises to 800 °C, the intensity of the unknown diffraction peak is stronger than that of BSCO-8, which further shows that the deposition process of BSCO-7 is better. At the same time, the substrate temperature should be controlled between 780 and 790 °C when the thin film is prepared by annealing at 510 °C for 2 h. 

**Figure 4:**
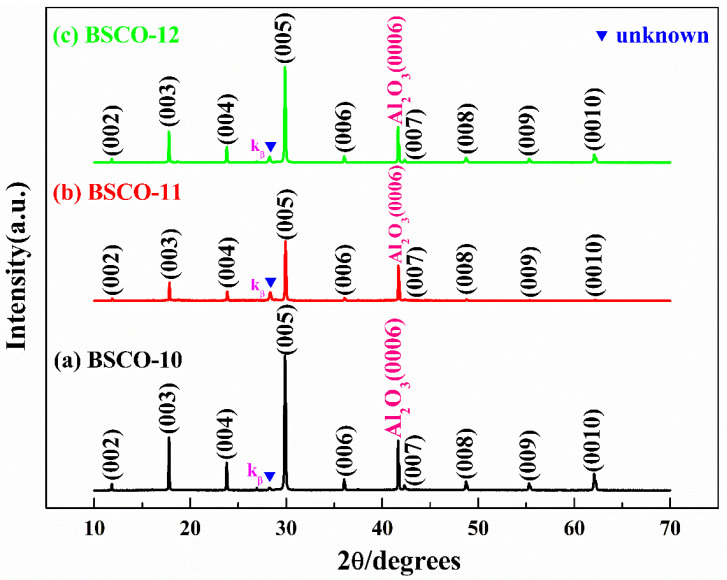
XRD patterns of thin films prepared by post-annealing: (**a**) BSCO-10, (**b**) BSCO-11 and (**c**) BSCO-12.

Figure 4a–c are the XRD patterns of BSCO-10~BSCO-12. BSCO-10~BSCO-12 thin films also adopt the post-annealing process. The annealing time is extended to 3 h to research whether the unknown diffraction peak can be eliminated by further extending the annealing time. From Figure 4a–c, it can be seen that the unknown diffraction peak still exists when the annealing time is extended to 3 h, and the intensity of the impurity phase is equivalent to that of BSCO-7~BSCO-9 thin film, which indicates that the impurity phase cannot be completely eliminated by extending the annealing time to 3 h.

### 3.3. Non-Annealing

**Figure 5:**
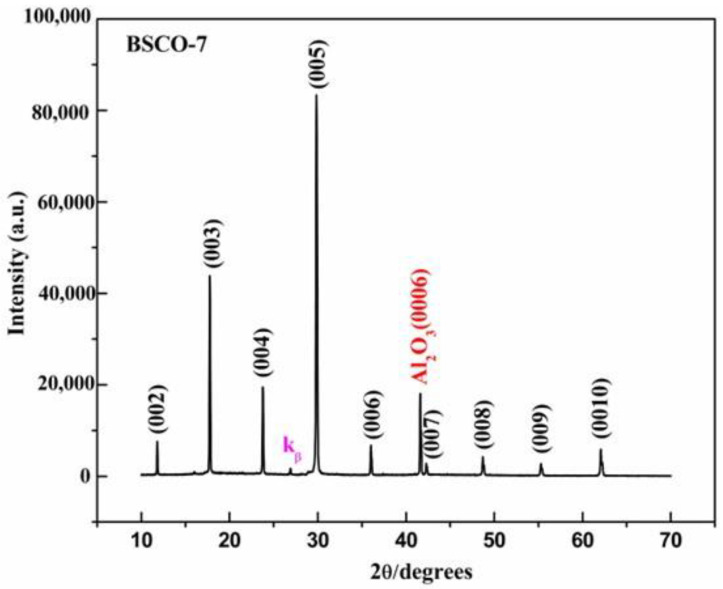
XRD patterns of thin films prepared by non-annealing.

In order to prepare pure-phase BSCO thin film, considering the above two preparing processes, the non-annealing process is adopted, namely, the thin film is cooled down to room temperature immediately after deposition. Table 3 shows the deposition process of BSCO thin film by non-annealing. Figure 5 is the XRD pattern of BSCO-13. All the diffraction peaks can be characterized as the diffractions of BSCO except for the substrate peak, which means that a pure-phase BSCO thin film has been prepared on the Al_2_O_3_ (0001) substrate by the PLD technique. Furthermore, the diffraction peaks are sharp, implying that the BSCO thin film has good crystallinity. Good crystalline quality is beneficial to obtain strong peak voltage. It is important to note that all the diffraction peaks of BSCO thin film are (00l), indicating that thin film is obviously c-axis preferred orientation. 

### 3.4. LITV Effect

**Figure 6:**
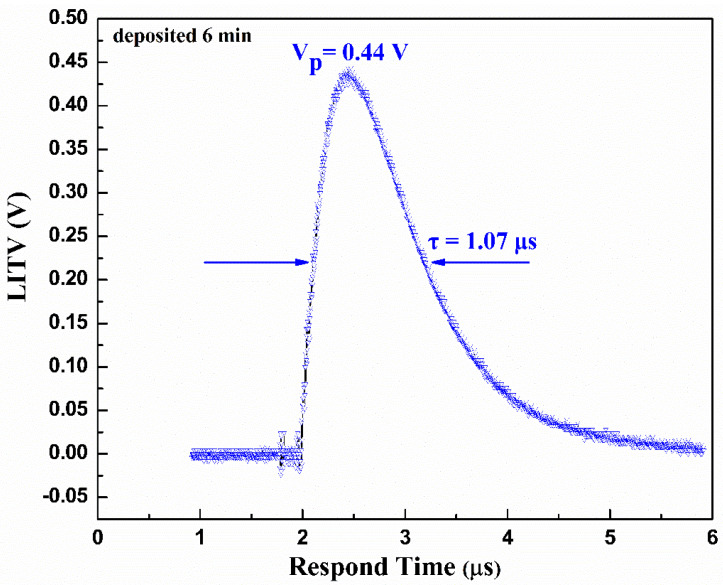
The LITV signal of Bi_2_Sr_2_Co_2_O_y_ thin film on 10° tilting Al_2_O_3_ (0001) substrate with deposition time of 6 min.

Figure 6 illustrates the LITV signal of BSCO thin film on 10° tilting the Al_2_O_3_ (0001) substrate with a deposition time of 6 min at room temperature. The single-pulse laser energy on the thin film is 246 mJ. It can be seen that there is an obvious LITV signal in BSCO thin film. The crystal structure of Bi_2_Sr_2_Co_2_O_y_ is composed of a conductive CoO_2_ layer and an insulating rock-salt-type layer. These two layers possess the same a- and c-axis lattice parameters and β angles but different b- axis lattice parameters, causing a misfit along the b direction [27]. This layered crystal structure leads to high anisotropic electronic properties, such as the ab-plane Seebeck coefficient S_ab_ being much larger than that of along c-axis S_c_. The S_ab_–S_c_ is about several tens of μV/K [28,29]. When the BSCO thin film is irradiated by a pulsed laser beam, the surface of the thin film absorbs the laser energy, the difference in temperature is come into being between the bottom and the top of the thin film, resulting in a temperature gradient perpendicular to the thin film surface. Owing to the highly anisotropic Seebeck coefficients between ab-plane and along c-axis, the temperature gradient in the ab-plane and along the c-axis is different, resulting in the difference in electron mobility. Ultimately, the electrons transfer to the indium electrodes, and the transverse thermoelectric voltage is detected. The peak voltage (V_p_) is 0.44 V, which is much stronger than the V_p_ achieved in LSCO (V_p_ is 0.15 V) [7], LCMO (V_p_ is 0.16 V) [10], and ZnO (V_p_ is 0.27 V) [30] thin films. The response time (full width at half maximum) is 1.07 μs. This result indicates that BSCO thin film can be applied to manufacture the probe of detectors due to large peak voltage and fast response speed.

**Figure 7:**
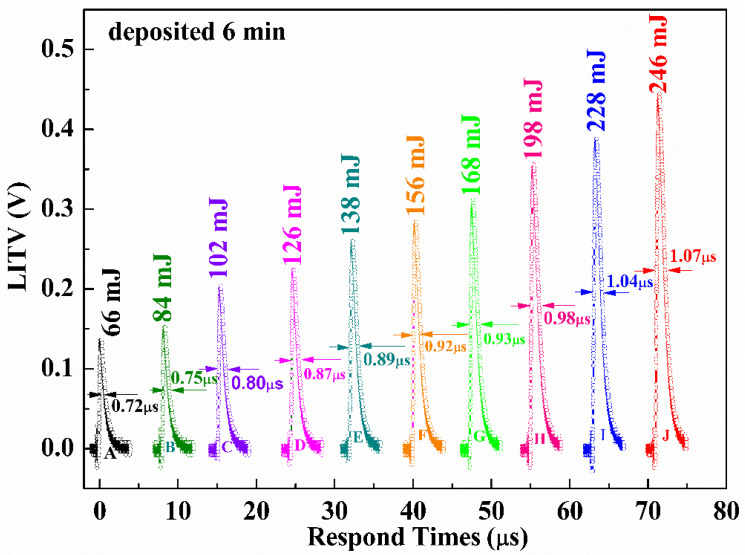
The LITV signals of Bi_2_Sr_2_Co_2_O_y_ thin film on 10° tilting Al_2_O_3_ (0001) substrate with different single-pulse laser energies.

Figure 7 presents the LITV signals of BSCO thin film with different single-pulse laser energies. The single-pulse laser energies are 66, 84, 102, 126, 138, 156, 168, 198, 228 and 246 mJ, respectively. It can be seen that the peak voltage increases from 0.14 to 0.44 V when the single-pulse laser energy is increased from 66 to 246 mJ. In other words, the peak voltage enhances with the increase of the single-pulse laser energies. Furthermore, the response time slows down from 0.72 to 1.07 μs with an increase of single-pulse laser energies. The surface temperature of the film increases with the incident laser energy, resulting in the increase in the temperature gradient between the bottom and the top of the thin film, which slows down the falling edge of the LITV signal, so the response time increases. The peak voltage and response time for samples corresponding to different single-pulse laser energies are summarized in Table 4.

**Figure 8:**
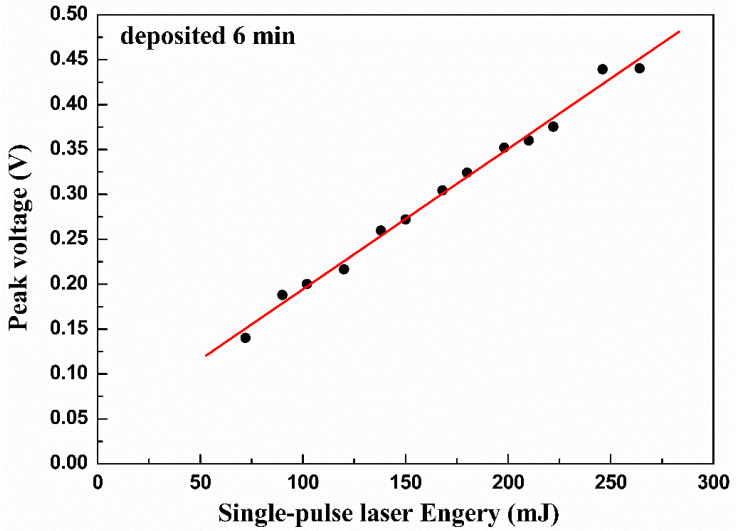
The linear relationship between the peak voltages and the single-pulse laser energy.

The relationship between the peak voltage and the single-pulse laser energy is demonstrated in Figure 8. There is a good linear relationship between peak voltage and single-pulse laser energy. Based on the atomic layer thermopile model, the time-dependent LITV effect can be defined as the following equation [31]:

(1) $$U\left(t\right)=\frac{\alpha_{0}El\sin\left(2\alpha \right)}{4d\rho c_{0}\sqrt{\pi Dt}}\left(S_{ab}-S_{c}\right)\left(e^{{-\delta }^{2}/4Dt}-e^{{-d}^{2}/4Dt}\right)$$

where *t* is the time, $\alpha_{0}$ is the absorption coefficient of thin film, *E* is the single pulse laser energy, *l* is the effective length of film irradiated by laser, *α* is the tilt angle of substrate, *d* is thin film thickness, *ρ* is the density of thin film, $c_{0}$ is the specific heat capability of thin film, *D* is the thermal diffusion coefficient of thin film, *S_ab_* is the Seebeck coefficient in *ab*-plane, *S_c_* is the Seebeck coefficient along *c*-axis and *δ* is the penetration depth of laser. It is known from Equation (1) that the voltage is proportional to the single-pulse laser energy; therefore, the V_p_ enhances linearly with the increase of the single-pulse laser energies. Our experimental result is consistent with Equation (1), as indicated in Figure 8. It is worth noting that appropriate single-pulse laser energy is not the higher the better, as there exists an upper limit value. This is because too-high single-pulse laser energy will result in the presence of conditions with temperatures too high on the surface of thin film and thus lead to its irreversible damage. Hence, the upper limit value of single-pulse laser energy should be controlled at a value that avoids the damage of thin film during the experiment of testing the LITV signal. In our experiment of testing the LITV signal, the upper limit value of single-pulse laser energy is 246 mJ. When the incident single-pulse laser energy exceeds 246 mJ, the film is damaged and the LITV signal cannot be detected.

**Figure 9:**
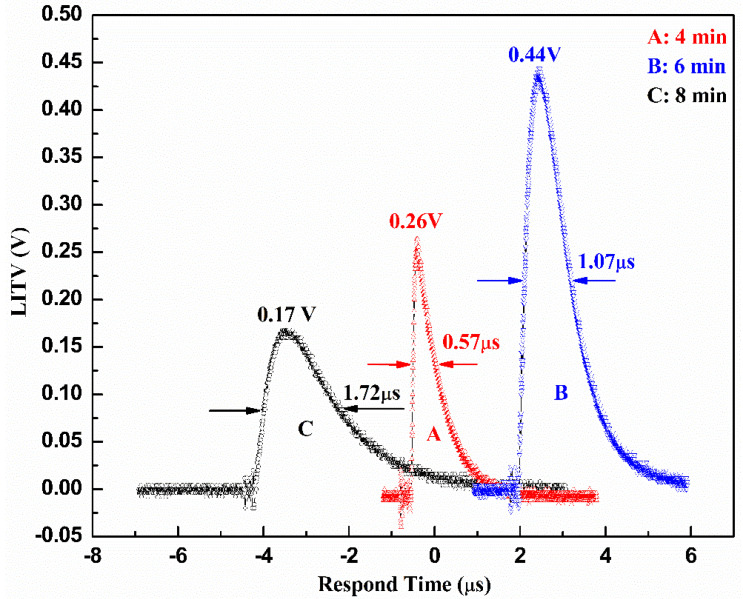
The LITV signals of Bi_2_Sr_2_Co_2_O_y_ thin films on 10° tilting Al_2_O_3_ (0001) substrate at different deposition times.

In order to comprehend the mechanism of the LITV effect better, the LITV signals of the BSCO thin films prepared at different deposition times are measured. The experimental conditions are the same as those deposited for 6 min. Figure 9 displays the LITV signals of BSCO thin films when deposition time is 4, 6 and 8 min, respectively. It is estimated that the thickness of these three films is about 240, 360 and 480 nm, respectively, on the basis of film growth rate (10 Å/S). It is clear that the peak voltage enhances with increasing deposition time from 4 min to 6 min, reaches the maximum value, 0.44 V and then decreases with deposition time. That is to say, an optimal deposition time corresponding to a maximum peak voltage value, does exist. This tendency of the deposition time dependence of peak voltage in BSCO thin film is consistent with that in La_0.5_Sr_0.5_CoO_3_ thin film [7] and SrTi_1−*x*_Nb*_x_*O_3_ thin film [14], when these films are irradiated by single-pulse laser. In addition, the response time slows down from 0.57 μs to 1.72 μs with increasement of deposition time, which is in agreement with Equation (1). The results of peak voltage and response time for samples grown at different times are listed in Table 5.

## 4. Conclusions

In summary, a high-quality Bi_2_Sr_2_Co_2_O_y_ thin film was grown on an Al_2_O_3_ single crystal substrate which is cut with a 10° tilting angle along the (0001) direction through the pulsed laser deposition technique with a non-annealing process. The LITV signals were detected in Bi_2_Sr_2_Co_2_O_y_ thin films. It was found that the peak voltage signal was strong, reaching 0.44 V and the response time was 1.07 μs. There was a good linear relationship between peak voltage and the single-pulse laser energy. All the above results suggest that Bi_2_Sr_2_Co_2_O_y_ thin films used as laser energy/power detectors will be more and more competitive due to high peak voltage and fast response speed. In addition, since the laser-induced thermoelectric voltage effect originates from the anisotropy of the Seebeck coefficient, it can be extended to other types of materials as long as the materials have an anisotropic Seebeck coefficient. 

## Figures and Tables

**Table 1:** In situ annealing process.

Samples	Substrate Temperature (°C)	Flowing Oxygen Pressure (Pa)	Deposition Time (min)	Pulsed Laser Energy (mJ/Pulse)	Annealing Time (min)
BSCO-1	820	100	8	300	30
BSCO-2	820	60	8	300	30
BSCO-3	850	60	8	300	30

**Table 2:** Post-annealing process.

Samples	Substrate Temperature (°C)	Flowing Oxygen Pressure (Pa)	Deposition Time (min)	Pulsed Laser Energy (mJ/Pulse)	Annea-Ling Time (min)	Annealing Temperature (°C)
BSCO-4	760	60	8	300	60	510
BSCO-5	800	60	8	300	60	510
BSCO-6	805	60	8	300	60	510
BSCO-7	780	60	8	300	120	510
BSCO-8	795	60	8	300	120	510
BSCO-9	800	60	8	300	120	510
BSCO-10	780	60	8	300	180	510
BSCO-11	795	60	8	300	180	510
BSCO-12	800	60	8	300	180	510

**Table 3:** Non-annealing process.

Sample	Substrate Temperature (°C)	Flowing Oxygen Pressure (Pa)	Deposition Time (min)	Pulsed Laser Energy (mJ/Pulse)
BSCO-13	790	60	8	300

**Table 4:** The peak voltage and respond time of different single-pulse laser energy.

Samples	Single-Pulse LASER Energy (mJ)	Peak Voltage (V)	Respond Time (μs)
A	66	0.14	0.72
B	84	0.15	0.75
C	102	0.20	0.80
D	126	0.22	0.87
E	138	0.26	0.89
F	156	0.28	0.92
G	168	0.31	0.93
H	198	0.36	0.98
I	228	0.39	1.04
J	246	0.44	1.07

**Table 5:** Peak voltage and response time of thin films at different deposition times.

Samples	Deposition Time (min)	Peak Voltage (V)	Respond Time (μs)
A	4	0.26	0.57
B	6	0.44	1.07
C	8	0.17	1.72

## Data Availability

The datasets generated and/or analyzed during the current study are available from the corresponding author upon reasonable request.
